# Nucleotide variants of the cancer predisposing gene *CDH1* and the risk of non-syndromic cleft lip with or without cleft palate

**DOI:** 10.1007/s10689-014-9727-2

**Published:** 2014-05-17

**Authors:** Kamil K. Hozyasz, Adrianna Mostowska, Piotr Wójcicki, Agnieszka Lasota, Barbara Offert, Adam Balcerek, Izabella Dunin-Wilczyńska, Paweł P. Jagodziński

**Affiliations:** 1 Department of Paediatrics, Institute of Mother and Child, 17a Kasprzaka Street, 01-211 Warsaw, Poland; 2 Department of Biochemistry and Molecular Biology, Poznan University of Medical Sciences, Poznan, Poland; 3 University Clinic of Medical Academy, Wroclaw, Poland; 4 Department of Plastic Surgery, Specialist Medical Center, Polanica Zdroj, Poland; 5 Department of Jaw Orthopaedics, Medical University of Lublin, Lublin, Poland; 6 Department of Paediatric Surgery, Institute of Mother and Child, Warsaw, Poland

**Keywords:** NSCL/P, CDH1, Cancer

## Abstract

The *CDH1* gene plays an important role during carcinogenesis and craniofacial morphogenesis. Germline mutations in this gene have been described in families presenting syndromic diffuse gastric cancer and orofacial clefts. The aim of this study was to evaluate the association between nucleotide variants of *CDH1* and the risk of non-syndromic cleft lip with or without cleft palate (NSCL/P). Six single nucleotide polymorphisms (SNPs) of the *CDH1* gene (rs16260, rs9929218, rs7186053, rs4783573, rs16958383, and rs1801552) were genotyped using the TaqMan SNP genotyping assays in 250 patients with NSCL/P and 540 controls from the Polish population. Comparison between patient and control groups showed that the *CDH1* rs1801552 variant, under the assumption of recessive model, was associated with a two-fold decrease in the risk of NSCL/P (OR_TT vs CT + CC_ = 0.481, 95 % CI 0.281–0.824, *p* = 0.007). This association remained statistically significant even after the multiple testing correction. No significant associations with NSCL/P risk were found for the other five tested SNPs. We found a strong association between the cancer predisposing gene *CDH1* and the risk of NSCL/P in the Polish population. This result, together with previous observations of co-occurrence of orofacial clefts and a variety of cancer types, suggests the need for replication studies testing rs1801552 in NSCL/P cohorts with a known cancer history.

## Introduction

Non-syndromic cleft lip with or without cleft palate (NSCL/P, OMIM 119530) is the most common facial birth defect and can cause problems with feeding, hearing, speaking, emotional development and social integration during growth [1]. Orofacial clefts have a complex etiology with both genetic and environmental factors contributing to the condition [1, 2]. Well known candidate genes, which are correlated with the risk of NSCL/P in various populations, include *IRF6*, *VAX1* and the 8q24 locus [1, 3]. However, their nucleotide variants do not account for all observed NSCL/P cases, emphasizing the need for identifying new genetic factors associated with NSCL/P.

Several studies have reported an association between orofacial clefts and cancer [4–6]. It has been proposed that these disorders may occasionally have a common etiology [4, 6]. Factors that have been suspected to be at the basis of these associations are polymorphic variants in genes involved in cell-to-cell adhesion and cell motility [4, 6]. Gastric cancer is the fourth most common malignancy and the second leading cause of death due to cancer worldwide [7]. The vast majority of gastric cancers are sporadic, although a small number, 3–5 % of them, is caused by an autosomal dominant inherited trait. The majority of families with autosomal dominant gastric carcinoma have the diffuse, poorly differentiated morphologic subtype (*linitis plastica*) which is referred to as hereditary diffuse gastric cancer (HDGC) [8, 9]. There is also emerging evidence for an increased risk of lobular breast cancer and colon cancer in HDGC families [8, 9]. Approximately 25–48 % of the individuals with HDGC have an autosomal dominant inherited germline *CDH1* mutation or large deletion affecting the *CDH1* locus. The *CDH1* gene, located on chromosome 16q22.1, encodes E-cadherin (OMIM: *192090), which is involved in epithelial calcium-dependent cell-to-cell adhesion [10, 11]. Currently, there are no reliable clinical screening methods for early detection of HDGC, which is located submucosally. Prophylactic total gastrectomy is the recommended form of management for individuals over 20 years of age carrying a *CDH1* mutation, because of their 80 % lifetime risk of developing gastric cancer and the limited value of surveillance modalities [8, 9]. The increased occurrence of cleft lip/cleft palate in HDGC patients with a *CDH1* mutation was suggested by Frebourg et al. [12] and supported by Kluijt et al. [13], who have described 4/58 (7 %) *CDH1* germline mutation carriers with orofacial cleft in the Dutch study for familial cancer. In France, approximately 6 % of registered *CDH1* germline mutation carriers have an orofacial cleft [14]. Recently, the incorporation of a family history of orofacial cleft was suggested into the new HDGC-defining criteria [14].

The most widely studied polymorphic variant of the *CDH1* gene is rs16260 (−160C>A), located upstream of the transcriptional start site of the *CDH1* promoter [11]. It has been shown that the −160A allele decreases the transcription efficiency of the *CDH1* gene [15, 16]. Meta-analyses suggest that rs16260 may be associated with the risk of colorectal cancer (CRC) in Western populations and sporadic gastric carcinoma among Caucasians, but not among Asians [11, 17]. Interestingly, in the study of Zhan et al. [18], genotypes of the *CDH1* rs16260 variant contributed to the risk of diffuse gastric cancer in ethnic Han Chinese. In the same population, Song and Zhang [19] found no significant association between NSCL/P and rs16260, whereas an association was observed for isolated cleft palate. The study of Rafighdoost et al. [20] revealed significant impact of the rs16260 AC and AA genotypes on NSCL/P risk in Iranians. Furthermore, two functionally missense germline mutations of *CDH1* were recently identified in 5.2 % (3/58) of children of European descent with NSCL/P [21]; however, there are unfortunately no studies assessing the association between rs16260 and NSCL/P in Europeans. Identification of clinical and molecular markers of individuals at increased risk of developing sporadic and familial gastric cancer are needed. There is strong evidence showing a positive association between the mislocalized, diminished or absent E-cadherin immunoreactivity and gastric cancer, and therefore, whenever possible, it is important to define the pathogenicity, as well as phenotypic manifestations, of *CDH1* variants [8, 14, 18]. Individuals identified as carrying specific genetic variants can be the target of more aggressive screening programs [4]. Development of early screening protocols for patients with orofacial clefts may lead to early stage diagnoses of cancer.

Therefore, we conducted an association study to determine whether common nucleotide variations in the *CDH1* gene may contribute to the risk of NSCL/P in the Polish population.

## Materials and methods

### Patients and controls

Peripheral blood samples from 250 unrelated subjects with NSCL/P were obtained from the Department of Paediatrics and Paediatric Surgery at the Institute of Mother and Child in Warsaw, the Department of Plastic Surgery Specialist Medical Center in Polanica Zdroj, and from the Department of Jaw Orthopaedics at the Medical University of Lublin. Eligibility to the patient group was ascertained from detailed medical records. Patients (aged 1–15 years) were examined by experienced medical geneticists. The non-syndromic designation was based on diagnosis of isolated CL/P with no other apparent cognitive and structural anomalies. Individuals with cleft palate only (CPO) were excluded from the study. The control group was composed of 540 healthy individuals with no family history of cleft lip and palate or other congenital anomalies. All participants were Caucasians of Polish origin born in Poland. DNA was isolated from peripheral blood lymphocytes by a salting-out extraction procedure. The experiments were approved by the local Ethics Committee at the Poznan University of Medical Sciences. Written and oral consent was obtained from the legal guardians of all the participants.

### SNP selection and genotyping

**Fig. 1:**
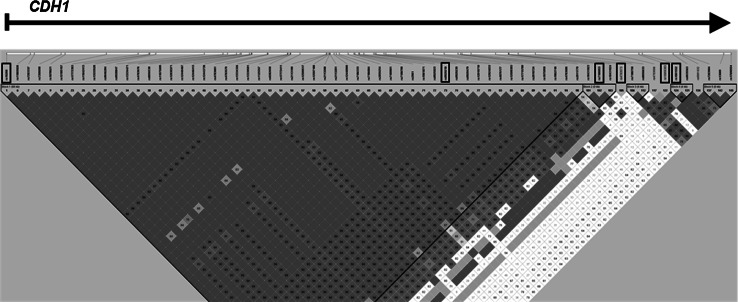
The linkage disequilibrium (LD) *plot* of HapMap SNPs within the *CDH1* region. The *plot* was generated using the genotype data from HapMap CEU samples and the Haploview 4.0 software (Broad Institute, Cambridge, MA). The names of the examined SNPs are enclosed in *boxes*. The *numbers* in the *squares* indicate percentage of LD between a given pair of SNPs (D’ values). *Blue squares* are non-informative. (Color figure online)

Single nucleotide polymorphisms (SNPs) in *CDH1* were identified from public databases including the NCBI dbSNP database (http://www.ncbi.nlm.nih.gov/projects/SNP/) and the HapMap Genome Browser (http://hapmap.ncbi.nlm.nih.gov/), and related literature. A final set of 6 SNPs was selected based on minor allele frequency (MAF) over 15 % in the Caucasian population, the gene-linkage disequilibrium (LD) patterns, and functional significance of SNPs. The LD pattern and the structure of haplotype blocks across the *CDH1* gene were determined using genotype data from the HapMap database and Haploview 4.0 software package (http://www.broad.mit.edu/mpg/haploview/). The plot of the pairwise LD between SNPs in *CDH1* is presented in Fig. 1. Characteristics of SNPs selected for the final analysis are presented in Table 1. Genotyping was carried out on the LightCycler 480 system (Roche Diagnostics, Mannheim, Germany) using pre-designed and custom TaqMan SNP Genotyping Assays according to the manufacturer’s instructions provided by Applied Biosystems (Applied Biosystems, Foster City, CA). Data analysis was performed using the Endpoint Analysis module of LightCycler 480 Software 1.5. For quality control, the genotyping analysis was blinded to the subject’s case–control status. In addition, approximately 10 % of the randomly chosen samples were re-genotyped.

**Table 1:** Characteristics of the *CDH1* polymorphisms genotyped in the dataset

rs no.	Chromosomal position^a^	SNP function	Alleles^b^	MAF^c^
rs16260	chr16:68771034	nearGene-5	A/C (FWD)	0.29
rs9929218	chr16:68820946	intron	A/G (FWD)	0.29
rs7186053	chr16:68839293	intron	A/G (FWD)	0.45
rs4783573	chr16:68840588	intron	A/G (FWD)	0.27
rs16958383	chr16:68857000	intron	A/G (FWD)	0.19
rs1801552	chr16:68857441	cds-synon (Ala692Ala)	C/T (FWD)	0.36

*FWD* forward, *REV* revers

^a^Accordnig to the February 2009 human reference sequence (GRCh37)

^b^According to the single nucleotide polymorphism database (dbSNP); Underline denotes the minor allele in the control samples

^c^ *MAF* minor allele frequency calculated from the control samples

### Statistical analysis

For each SNP, the Chi square test (χ^2^) for Hardy–Weinberg equilibrium (HWE) and minor allele frequency (MAF) were computed among both NSCL/P patients and controls. The differences in allele and genotype frequencies between patients and controls were determined using standard χ^2^ and Fisher exact tests. SNPs were tested for association with NSCL/P using the Cochran-Armitage trend test. Odds Ratios (ORs) with 95 % Confidence Intervals (95 % CIs) were used to assessed the strength of the association. The dominant and recessive models were analyzed. A statistical adjustment for multiple comparisons was accomplished by using the Bonferroni correction. Statistical significance was interpreted as *p* values <0.00833. Pair-wise LD was calculated as both D’ and r^2^ for all tested SNPs using Haploview software. Haplotype association testing was performed using the UNPHASED 3.1.5 program with the following analysis options: all window sizes, full model and uncertain haplotype option [22]. Statistical significance was assessed using the 1,000-fold permutation testing.

## Results

### Single-marker association analysis

All tested SNPs did not show deviation from HWE in both patients and controls (*p* > 0.05). The MAF for tested markers was at least 19 %. The genotyping results, OR and 95 % CI calculations for the 6 tested SNPs of *CDH1* are reported in Table 2. Under assumption of a recessive model (TT vs CC + CT, where T is the minor allele), the calculated OR for rs1801552 was 0.481 (95 % CI 0.281–0.824, *p* = 0.0016). This result was statistically significant even after Bonferroni correction (*p* < 0.00833). The OR calculated for individuals with the combined TT and CT genotypes of the rs1801552 polymorphism compared to CC homozygotes (dominant model) was 0.955 (95 % CI 0.705–1.293), but the result was not statistically significant (*p* = 0.764). For rs1801552, the genotype frequencies showed a significant difference between cases and controls (*p* = 0.021). For the remaining analyzed *CDH1* SNPs, there was no evidence for both allelic and genotypic association with the risk of orofacial clefts (Table 2). Analysis of pair-wise LD between the investigated *CDH1* SNPs revealed that rs1801552 was not correlated with other variants. D’ and r^2^ values, calculated from the genotype data of the control samples, ranged from 0.033 to 1.000 and 0.001 to 0.129, respectively (Table 3).

**Table 2:** Association of polymorphic variants of CDH1 with the risk of NSCL/P

rs no.	Alleles^a^	MAF^b^	Genotypes cases^c^	Genotypes controls^c^	p_trend_ value	p_genotypic_ value	p_allelic_ value	OR_dominant_ (95 % CI)^d^; *p* value	OR_recessive_ (95 % CI)^e^; *p* value
rs16260	A/C	0.29	22/97/131	49/213/278	0.811	0.970	0.807	0.964 (0.714–1.301); 0.810	0.967 (0.571–1.638); 0.900
rs9929218	A/G	0.29	22/102/126	48/222/270	0.921	0.995	0.921	0.984 (0.729–1.328); 0.917	0.989 (0.583–1.678); 0.967
rs7186053	A/G	0.45	48/118/84	111/261/168	0.480	0.766	0.474	0.893 (0.648–1.229); 0.485	0.918 (0.630–1.340); 0.659
rs4783573	A/G	0.27	18/95/137	40/210/290	0.787	0.959	0.785	0.957 (0.708–1.293); 0.774	0.970 (0.544–1.728); 0.917
rs16958383	A/G	0.19	8/78/164	23/155/362	0.930	0.637	0.929	1.066 (0.777–1.464); 0.690	0.743 (0.328–1.686); 0.476
**rs1801552**	**C/** **T**	0.36	**18/125/107**	**75/240/225**	**0.126**	**0.021**	**0.129**	**0.955 (0.705–1.293); 0.764**	**0.481 (0.281–0.824); 0.007**

Statistically significant results are highlighted in bold (*p* < 0.00833—Bonferroni correction)

^a^Underline denotes the minor allele in the control samples

^b^ *MAF* minor allele frequency calculated from the control samples

^c^The order of genotypes: dd/Dd/DD (d is the minor allele in the control samples)

^d^Dominant model: dd + Dd versus DD (d is the minor allele)

^e^Recessive model: dd versus Dd + DD (d is the minor allele)

**Table 3:** Linkage disequilibrium between markers of the CDH1 gene in the control samples

	rs16260	rs9929218	rs7186053	rs4783573	rs16958383	rs1801552
rs16260		0.982	0.909	0.851	0.829	0.131
rs9929218	0.934		0.890	0.831	0.834	0.139
rs7186053	0.413	0.409		0.734	0.541	0.112
rs4783573	0.108	0.106	0.16		0.322	0.033
rs16958383	0.064	0.066	0.054	0.065		1.000
rs1801552	0.012	0.014	0.006	0.001	0.129	

D′ above diagonal

r^2^ below diagonal

### Haplotype analysis

Haplotype analysis of the studied *CDH1* polymorphisms did not show SNP combinations associated with the risk of NSCL/P (Table 4). The lowest global *p* = 0.087 was observed for haplotype composed of the rs9929218, rs7186053, rs4783573 and rs16958383 SNPs (Table 4). However, this result was not statistically significant when permutations were used to generate empiric *p*-values. The empirical 5 % quintile of the best *p* value after 1,000 permutations was 0.003295.

**Table 4:** Results of haplotype analysis of the CDH1 gene in patients with NSCL/P

Polymorphisms	Χ^2^	Global *p* value
rs16260_rs9929218	4.736	0.192
rs9929218_rs7186053	0.439	0.508
rs7186053_rs4783573	2.230	0.135
rs4783573_rs16958383	0.239	0.971
rs16958383_rs1801552	2.537	0.281
rs16260_rs9929218_rs7186053	2.156	0.541
rs9929218_rs7186053_rs4783573	4.654	0.199
rs7186053_rs4783573_rs16958383	6.507	0.089
rs4783573_rs16958383_rs1801552	5.674	0.339
rs16260_rs9929218_rs7186053_rs4783573	8.512	0.290
rs9929218_rs7186053_rs4783573_rs16958383	11.060	0.087
rs7186053_rs4783573_rs16958383_rs1801552	8.309	0.306
rs16260_rs9929218_rs7186053_rs4783573_rs16958383	15.298	0.122
rs9929218_rs7186053_rs4783573_rs16958383_rs1801552	13.329	0.272
rs16260_rs9929218_rs7186053_rs4783573_rs16958383_rs1801552	18.936	0.272

Empirical 5 % quantile of the best *p* value: 0.003295

## Discussion

Abnormal *CDH1* expression has been linked to many human diseases, including tumors, nephrolithiasis, pre-eclampsia, and ectopic pregnancy [10, 11]. Although great advances have been achieved in gene identification for NSCL/P, the underlying molecular mechanisms remain obscure. Identifying the underlying etiology is crucial in improving prevention strategies and genetic risk counseling. Recent epidemiological findings point toward at least some shared genetic risk factors of NSCL/P and cancer [4, 5]. It is possible that several truly NSCL/P associated variations are hidden among the list of moderately significant SNPs. In this study, we assessed if polymorphic variants in the cancer predisposing gene *CDH1* are associated with NSCL/P in a sample from the Polish population. Our results suggest that the presence of the *CDH1* rs1801552 TT genotype is associated with a two-fold decreased risk for NSCL/P in the investigated population, but further studies with larger cohorts from different populations and taking into account family history of cancer are warranted. Unfortunately, there are no published association studies of rs1801552 and gastric cancer in Poland. In China, this polymorphic variant of *CDH1* has not achieved significant difference in its distribution between gastric cancer cases and controls [18]. The German study of Jacobs et al. [23] revealed a contribution of *CDH1* rs1801026, but not rs1801552, to a predisposition to the development of primary gastric diffuse large B-cell carcinoma. The impact of the rs1801552 polymorphism on CDH1 activity in tissues remains unclear. However, a significant difference in plasma CDH1 levels among carriers with different *CDH1* rs1801552 genotypes has not been reported [18]. Further in vitro and in vivo functional studies are needed to characterize the functional significance of this SNP. Loss of expression of E-cadherin leads to an increased ability of cells to invade neighboring tissues [10, 16]. Our findings are of interest especially in light of observations showing that individuals without a *CDH1* mutation that are presenting with tumors that have E-cadherin expression impairment, similar to that observed in *CDH1* germline mutation carriers with HDGC, may have a *CDH1* expression defect caused be either direct or indirect mechanisms targeting the *CDH1* genomic sequence [24]. In respect to this assumption, the reported simultaneous familial occurrence of NSCL/P and cancer could provide clues to consider *CDH1* rs1801552 as a potential marker of cancer susceptibility [4, 6].

Our study has not confirmed an association between rs16260 and NSCL/P, which is controversial [19, 20]. Our single-marker analyses also did not show any evidence of correlation between the remaining rs9929218, rs7186053, rs4783573, and rs16958383 *CDH1* variants and the risk of NSCL/P. The last two SNPs were previously tested as breast cancer susceptibility markers in China [25]. The *CDH1* rs16958383 has been found to have a borderline association with breast cancer in premenopausal, but not postmenopausal, women [25]. Recently, Ierodiakonou et al. [26] showed that rs7186053, rs4783573 and rs16958383 may contribute to airway remodeling and lung function in asthma patients using inhaled corticosteroids, however their impact on pulmonary cancer has yet to be elucidated.

Haplotype analysis has shown that the 4-SNP haplotype composed of rs9929218, rs7186053, rs4783573 and rs16958383 tends to be correlated with the risk of NSCL/P in our study population. Previously, rs9929218 was shown to have a borderline association with unilateral NSCL/P in Brazilians, who have undergone varying degrees of admixture with ancestors from widely divergent regions [27]. Taken together, the findings suggest this nucleotide variant may have different associations with NSCL/P etiology depending on specific ancestry. Evaluations of rs9929218 in relation to CRC risk and survival also showed significant associations [28, 29]. Thus, further examination of haplotypes in *CDH1*, including rs9929218, is needed to identify a biologically relevant cause.

Compaction of the preimplantation embryo is considered the earliest morphogenetic process essential for mammalian development. E-cadherin dependent filopodia attaching onto neighboring cells were demonstrated to control the shape changes necessary for compaction [30]. Leitra et al. [27] and Rafighdoost et al. [20] have suggested that *CDH1* may be a very reasonable candidate gene for NSCL/P. In mammals, CDH1 is required during early development and in establishing a proper connection between embryonic and maternal blood vessels [10]. It is expressed in the epithelium of the palate prior to and after shelf fusion. Epithelial to mesenchymal transition (EMT) is regarded an integral process in palatogenesis [27]. β-catenin, a member of the protein complex connecting cadherins to the actin cytoskeleton at adherens junctions, plays a crucial role in the onset and progression of EMT. At an early stage of transformation, the epithelial cell down-regulates its expression of E-cadherin, which frees the attachment of the cells from one another [10, 16]. It has been demonstrated that E-cadherin can compete with the transcriptional activity of the canonical WNT signaling pathway, increasing cell proliferation [31]. Interestingly, in our previous study we showed that a polymorphic variant of the gene encoding WNT3 and haplotype combinations of the *WNT3* SNPs were significantly correlated with the NSCL/P in the Polish population [32].

Although open to question, nutrients and nutrient-related transport factors have also been suggested as influential in orofacial cleft risk [1]. Cdh1 was shown to be involved in the mammalian endocycle, also known as endoreplicative cycle, and to participate in the differentiation of trophoblast stem cells to trophoblast giant cells during placental development [33].

The major limitations of this study are the sample size, which did not allow us to detect modest associations and interactions, and lack of data regarding personal and familial cancer history of participants. It would be interesting to explore the presented correlations in relation to cancer history. We must also note that the number of selected polymorphisms does not cover the *CDH1* gene fully and extensively. Allele frequencies are known to vary among different populations and different ethnic backgrounds. However, our study population was ethnically homogenous.

In summary, in this study we successfully genotyped six SNPs of *CDH1* in patients with NSCL/P and a properly matched control group. After adjusting for multiple comparisons, the *CDH1* rs1801552 variant was found to be associated with a protective effect against the risk of NSCL/P in the Polish population. Our results suggest the need for replication studies using the identified SNP associated with orofacial cleft susceptibility in NSCL/P cohorts with known cancer history in the participants and their families. If the nucleotide variant is again found to be associated with NSCL/P in a second population, then functional studies should be designed in an attempt to determine its biological role.
